# Nordic Walking Performance Analysis with an Integrated Monitoring System

**DOI:** 10.3390/s18051505

**Published:** 2018-05-10

**Authors:** Francesco Mocera, Giuseppe Aquilino, Aurelio Somà

**Affiliations:** Department of Mechanical and Aerospace Engineering, Politecnico di Torino, 10129 Torino, Italy; giuseppe.aquilino@studenti.polito.it (G.A.); aurelio.soma@polito.it (A.S.)

**Keywords:** Nordic walking, monitoring device, MEMS sensors, sport engineering

## Abstract

There is a growing interest in Nordic walking both from the fitness and medical point of views due to its possible therapeutic applications. The proper execution of the technique is an essential requirement to maximize the benefits of this practice. This is the reason why a monitoring system for outdoor Nordic walking activity was developed. Using data obtained from synchronized sensors, it is possible to have a complete overview of the users’ movements. The system described in this paper is able to measure: the pole angle during the pushing phase, the arms cycle frequency and synchronization and the pushing force applied to the ground. Furthermore, data from a GPS module give an image of the environment where the activity session takes place, in terms of the distance, slope, as well as the ground typology. A heart rate sensor is used to monitor the effort of the user through his/her Beats Per Minute (BPM). In this work, the developed monitoring system is presented, explaining how to use the gathered data to obtain the main feedback parameters for Nordic walking performance analysis. The comparison between left and right arm measurements allowed validating the system as a tool for technique evaluation. Finally, a procedure to estimate the peak pushing force from acceleration measurements is proposed.

## 1. Introduction

The regular practice of physical activity is an essential requirement for a healthy lifestyle regardless of age or social background. To maximize the benefits of a workout and avoid undesirable injuries, it is important to properly perform movements and to adapt the intensity to each individual characteristics. For this reason, in the last few years, several devices were proposed for the fitness market. Without a doubt, these devices help professional athletes improve their performance, but can also be used by non-professionals to maximize the benefits of their workouts.

This work focused on a particular sport discipline called Nordic walking. This is a modified version of standard walking, where the use of a specific pair of poles engages the upper body muscles, adding their contribution to the propulsive phase. In recent years, Nordic walking was promoted for its health benefits. The involvement of the upper body suggests a greater energy expenditure, thus a quicker improvement of the overall physical condition if compared to standard walking [1,2,3,4]. Some studies show that the use of these poles affects the weight distribution on joints and on the spinal column [5,6,7,8]. However, the medical and bioengineering scientific community are still investigating the real benefits of this technique, also for its possible therapeutic applications [9,10,11,12]. Although several studies seemed to confirm the advantages of practicing this discipline, others investigated if these are really attributable to the particular technique or not. The authors think that one of the possible reason of this wide variety of discussions may be related to how and where testing activities are conducted. Usually, these tests are designed for indoor activities [13,14,15,16,17,18,19] where high accuracy testing equipment also allow exploring physiological and biomechanical aspects in a controlled environment. Despite the high accuracy of these testing procedures, it is realistic to assert that in real workout sessions, the variability of the testing conditions may lead to different results. To help prove the feasibility of outdoor Nordic walking testing sessions, an integrated monitoring system was developed to measure performance in several environments. Using MEMS sensors [20,21,22,23], the system is able to identify the main parameters that characterize the proper movement technique giving feedback to the user about how well he/she performed. Acceleration and force measurements allow one to take into account the walking cycle in terms of frequency, effort of the upper body and proper technique execution. A specific processing algorithm was developed to estimate the angle between the pole and the ground during the pushing phase. Data coming from an integrated GPS system allow for user tracking during the workout session, giving access to information about the encountered slope and environmental conditions. There are several studies in the literature [24,25,26] that show how useful the application of sensors in movement analysis can be in ski-derived disciplines. Although several similarities can be found in ski and Nordic walking techniques, to the authors’ knowledge, there is low attention on the benefits of portable measurements platforms applied to the study of this discipline. Thus, this work wants to address the potential of the designed system as tool for Nordic walking performance evaluation.

In collaboration with Gabel, the Italian leader manufacturer of Nordic walking poles, with the help of a group of experts, a set of requirements for the new data acquisition system was defined in terms of weight specifications and parameters to be evaluated. In fact, the final system must have the lowest influence possible on the users’ movements in order to avoid undesirable compensations. The concept was designed to be the starting point of a possible commercial version. The load cell included in this prototype may not be present in the final version, considering that a proper analysis on acceleration data can lead to a good estimation of the peak during the pushing phase.

The paper has two main sections. The first one is mainly focused on the characteristics of the designed monitoring system, while the second moves the attention to the data output of the system and how to extract useful information from them to characterize the Nordic walking session. This experimental setup will allow gathering useful information about cycle and session performance that can be used in future works for kinematics and dynamic multibody-based analysis.

## 2. The Monitoring System

The monitoring of Nordic walking sessions is important both for the data scientific relevance and for the user experience. Several studies pointed out that there are differences between the results obtained in the literature, and it is widely accepted that testing equipment play a big role in this direction. They are designed for indoor applications and are usually cumbersome, forcing the users unconsciously to adapt their behavior to the testing machine. The monitoring system herein presented is thought to overcome some of the limitations of indoor equipment, but not to replace them. In fact, these equipment allow for better accuracy exploring physiological aspects that cannot be analyzed without specific tools.

### 2.1. Technical and Functional Requirements

In this sport discipline, the characteristics of the poles are crucial. They are designed to have a good intensity level of use of the upper body muscles without introducing a sensible fatigue contribution during the recovery phase. They are characterized by high stiffness, low weight and a specific geometric design to keep the impact on the users’ movements as low as possible [27].

**Figure 1:**
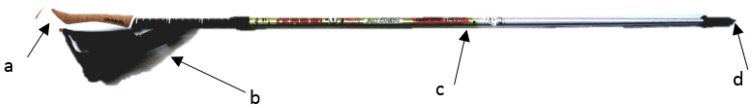
Nordic walking pole: (**a**) handle; (**b**) glove; (**c**) aluminum or carbon fiber body; (**d**) steel tip.

Nordic walking poles (Figure 1) are extremely light (180 g) to reduce the inertia contribution in the recovery phase. From the experience of several Nordic walking trainers, a set of requirements in terms of weight and position from the handle was stated to reduce the impact of the system on the movement. Moreover, to allow long outdoor sessions, the system had to be optimized in terms of energy consumption and acquisition performance.

Several studies in the literature agree on what are the main parameters to describe the Nordic walking technique. The most widely accepted are:

- the angle between the ground and the pole during the pushing phase;
- the time length of the cyclic movement;
- the pushing force when the pole is in contact with the ground.

In addition to these parameters, the heart rate can give a measure of the workout intensity, and GPS coordinates allow tracking the user path in terms of ground conditions (location) and slope. All these data were evaluated from raw sensor measurements. To correctly capture the dynamics of the movement, a minimum acquisition frequency of 15 Hz was fixed as the requirement of the acquisition board.

### 2.2. The Acquisition System

To build a device capable of collecting useful information about a Nordic walking session, a two-step design procedure was followed to define the board requirements:

- parameters identification;
- measurements definition.

The parameters identified in the previous section thanks to the literature review allowed stating the required measurements, thus the necessary hardware components. In Table 1, the main components used on the board and their main functionality are summarized.

**Figure 2:**
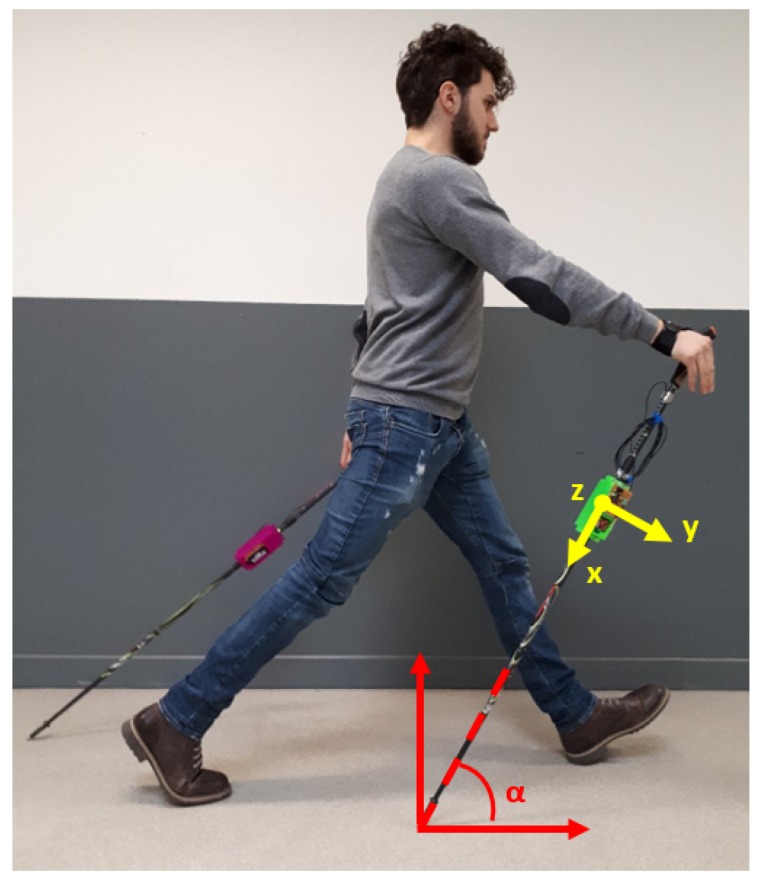
Accelerometer reference frame orientation.

**Figure 3:**
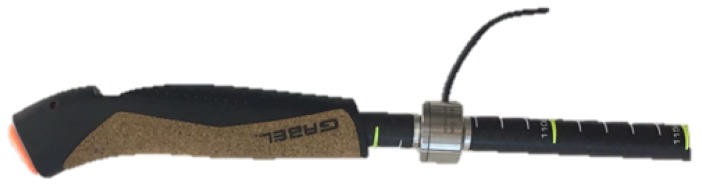
Integrated load cell.

- **Triaxial accelerometer**: From acceleration measurements of a properly-oriented accelerometer (Figure 2), it was possible to obtain several useful parameters: the cycle length, the mean angle of the pole during the pushing phase and, in some cases, an estimation of the impact force. For the specific application, the device was set up with a 10-bit resolution in the ±8-g range, using the standard sampling rate of 100 Hz. Note that the measured accelerations were digitally filtered by the device itself with a bandwidth of half the sampling rate (50 Hz in this case).
- **GPS**: From GPS coordinates, it was possible to track the user during the workout session. Knowing the geographic location, environmental characteristics such as the slope and ground properties can be evaluated. The device was set up to update position information every second. The availability of an absolute time source in the GPS message was considered as a simple way to synchronize data between the left and right device.
- **Load cell**: From axial force measurements (Figure 3), it was possible to understand how the user relies on poles for forward thrust motion. A higher mean value of the pushing force means higher effort of the upper body, which does not necessarily correspond to the correct approach. Poles should be used as a complementary driving force to the legs, not as the main pushing component. The axial load cell used in this application was characterized by a rated force of 500 N with a sensitivity of 1 mV/V/FS.
- **Heart rate sensor**: From a simple measurement of the heart Beats Per Minute (BPM), it was possible to have an idea of the user effort. This parameter can be used to study how the same session impacts users with different characteristics. From the medical point of view, using this parameter, it is possible to compare the performance of several patients and track their improvements over time.
- **Memory storage**: A storage solution was necessary to record all the acquired data coming from the sensors.
- **Micro-controller**: This was the device brain, which managed all the different sensors saving all the data on the storage unit with a certain format to simplify the post-processing phase.

**Figure 4:**
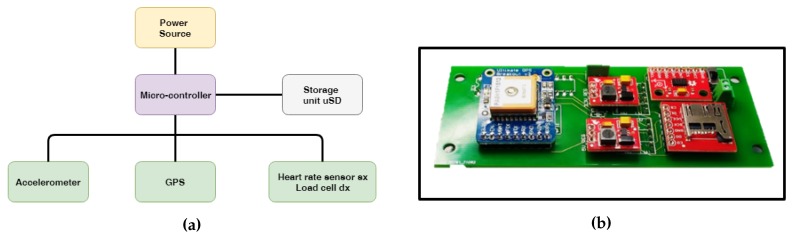
(**a**) Monitoring system layout; (**b**) data acquisition Printed Circuit Board (PCB).

In Figure 4a,b, the system functional layout and the actual data acquisition board are shown. As described in previous work [28,29], to allow long outdoor monitoring sessions, all of the system had to be optimized in terms of acquisition performance and energy consumption. The use of a properly-tuned energy harvester can extend the monitoring time using the available kinetic energy from the cyclic arm movement [30,31,32,33]. The energy consumption depends also on the performance requirements of the monitoring system. Through an optimization process, it was possible to reach the best trade off between performance and energy consumption. An acquisition frequency of 50 Hz was obtained with a total energy consumption in acquisition mode of 60 mW.

### 2.3. Post-Processing: Nordic Walking Data Analysis

The acquisition board was developed together with a proper post-processing tool to allow non-technical users to perform an easy evaluation of the main parameters. Data are stored by the microcontroller in the physical memory in a certain formatted text file to help the post-processing phase. The stored information is comprised of the following:

- latitude and longitude;
- date and time;
- acceleration measurements;
- user heart rate on the left pole;
- axial force on the right pole;
- internal microcontroller time for internal data synchronization.

As it will be discussed in the next sections, the raw data can be processed by the software tool in order to identify the parameters of interest, both as direct measurements on the time histories or as a combination between different data sources.

## 3. Measurements and Raw Data

The described system was capable of acquiring several measurements in a synchronized way. There was not a strict deterministic acquisition; however, the low dynamics of the phenomena involved allowed having rather accurate simultaneous measurements. The subject involved in these tests was one of the work authors, who successfully accomplished a Nordic walking training course. The subject is a young trained man (27 years old) with experience in several ski disciplines and trail running. These characteristics were considered sufficient as requirements for the system tester.

### 3.1. Acceleration Measurements

**Figure 5:**
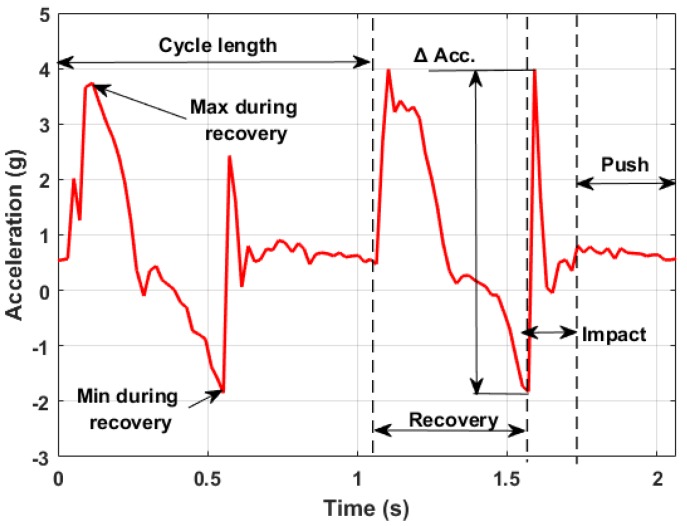
Typical longitudinal acceleration output measured by the acquisition system.

Acceleration measurements allowed characterizing each phase of the arm cyclic movement. In Figure 5, it is possible to see an usual output diagram of the acceleration measured along the longitudinal axis of the poles. The plot shows that the measured dynamic behavior is in agreement with what can be found in the literature [29]. The data measured along this axis allowed for several considerations. After the first contact between the pole tip and the ground, no other dynamic component is superimposed on the acceleration measured along this direction. Thus, since the chosen accelerometer measured also the static components of the gravity acceleration, it was possible to estimate the angle between the pole axis and the ground during the pushing phase. This can be defined as:

(1) $$cos\left(\phi \right)=\frac{a_{x}}{acos(90-\theta )}$$

where:

- *a* is the module of the acceleration vector a;
- $a_{x}$ is the a component along the *x* axis (pole longitudinal axis);
- θ is the angle between a and the *x*-*y* plane;
- ϕ is the angle between the projection of a on the *x*-*y* plane and the *x* axis.

However, in the specific application, the device was oriented in such a way that in its nominal position, the *x*-*y* plane was parallel to the body sagittal plane. This meant that nominally, θ was 90°, and thus:

(2) $$\phi =arcosa_{x}$$

During the exercise, the mentioned plane did not always remain strictly parallel to the body’s sagittal plane. Nevertheless, also without a perfect execution, the deviation from the nominal orientation of this plane was considered small enough to accept the approximation of the pole angle with the mentioned ϕ angle. It should be pointed out that also ground inclination may slightly affect the value of this angle. In this work, this effect was not considered since the main tests were conducted on plane fields. A more sophisticated version of the device may include in the future a 9DOF (Degrees Of Freedom) sensor in order to have additional information to evaluate the correct relative position between poles and ground.

Looking at the diagram shown in Figure 5, three main phases can be identified from the measured acceleration:

1. The **recovery phase** was where the poles essentially did not touch the ground and were in a generic roto-translational motion carried by the user. The pole lost contact with the ground and then was pulled forward so that a positive dynamic acceleration component was superimposed on the static gravity component. In the last part of this phase, the arm stopped its motion, pushing the pole towards the ground (negative acceleration values).
2. The **impact phase** was when the poles touched the ground. The impulsive nature of the contact translated into a high acceleration value reached in a small amount of time. Both the amplitude and the growth rate of this event strongly depend on soil characteristics and on the user technique.
3. The **pushing phase** was when the user pushed on the poles, already in contact with the ground, to move forward. The measured acceleration could not exceed 1 g in this condition since no relevant dynamic component was present in this phase.

The time duration of each cycle is a perfect measure of the repetitiveness of the movement. In the particular case shown above, the cycle lasts for about one second. The duration of each phase can also be considered to analyze the movement. In the specific case, the recovery phase lasted for about 50% of all of the cycle, while the impact and the pushing phase lasted 15% and 35% of the remaining cycle time.

**Figure 6:**
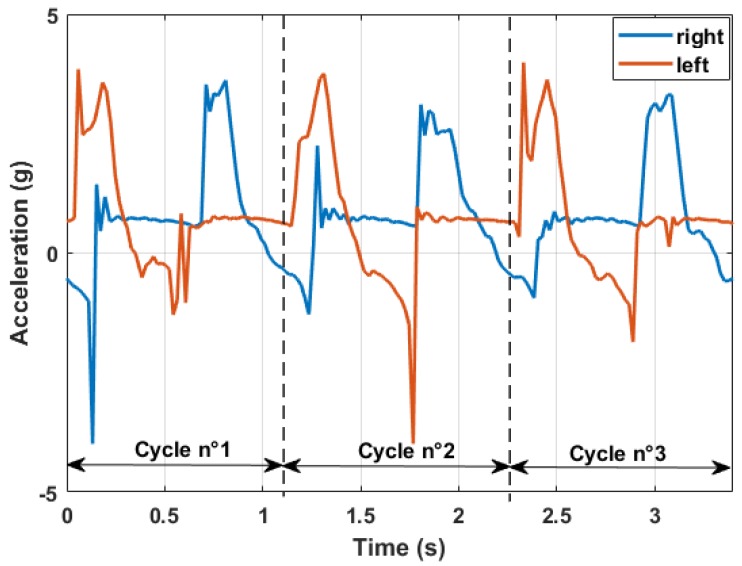
Acceleration data superimposition between each arm.

More interesting is the superimposition of the diagram coming from the left and right arm (Figure 6). Each pole had its own data acquisition system with a proper GPS sensor. The two devices were not able to communicate together, but the absolute time given by the GPS sensors and a proper synchronization algorithm allowed for a post-processing superimposition.

**Figure 7:**
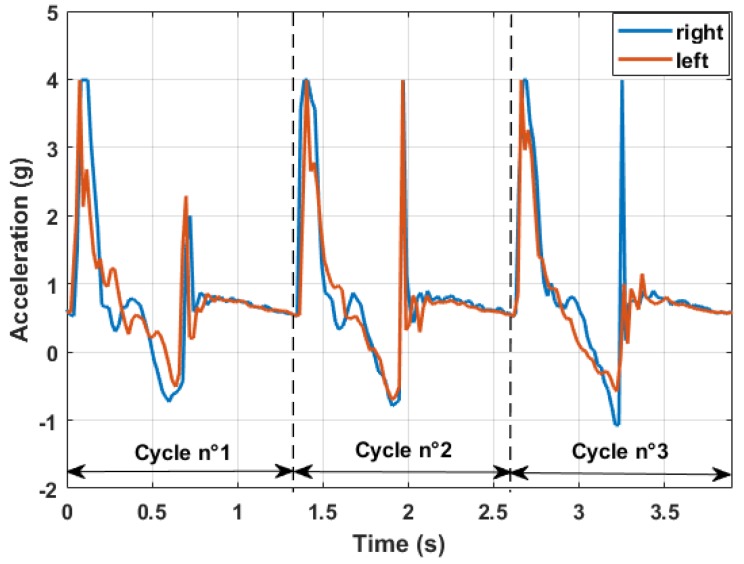
Parallel pushing technique.

Merging information coming from each pole, it was possible to establish how each arm was performing in terms of timing and most importantly of pushing angle. This comparison allows to characterize different Nordic walking styles as the one with the alternate pushing phase (shown above) or the parallel pushing variant shown in Figure 7. The latter technique is characterized by a longer cycle time length, leading to a more discontinuous movement that has to be properly compensated by the user’s core to maximize the effectiveness of the pushing phase.

### 3.2. Force Measurements

**Figure 8:**
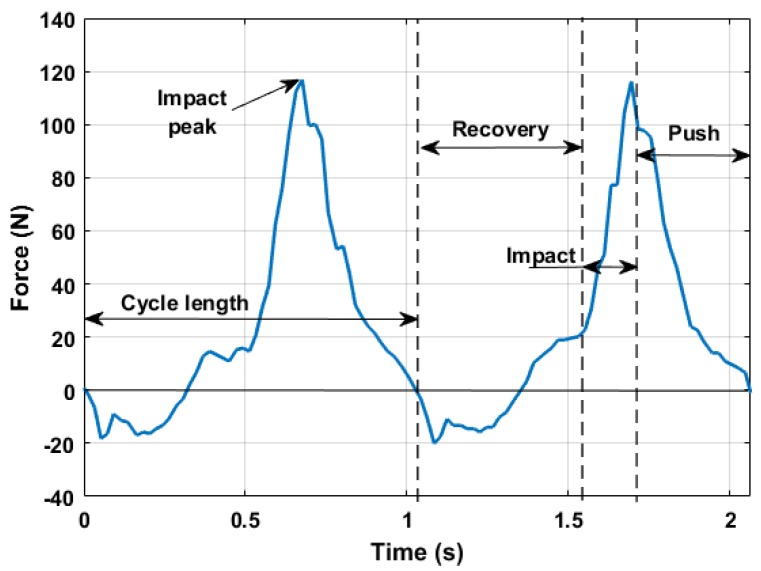
Force measurements along the longitudinal pole axis.

The integrated axial load cell allowed measuring the force along the pole longitudinal axis. In this analysis, the compliance effect of the pole was neglected due to the high stiffness of the carbon body of the equipped pole. Figure 8 shows results in agreement with indoor poling force measurements [13,14], especially as concerns the pushing force temporal distribution within the arm cycle and its peak values. In particular, it is possible to identify the three main phases of the cyclic movement:

1. The **recovery phase** when the force is negative (tension) and below a certain threshold;
2. The **impact phase** characterized by the increase of the pushing force;
3. The **pushing phase** when the force slowly decreases until the moment when the pole tip loses contact with the ground, starting a new cycle.

### 3.3. GPS Data

**Figure 9:**
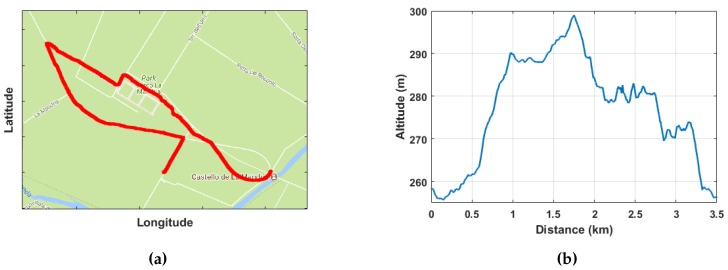
(**a**) GPS tracking; (**b**) altitude.

Through GPS coordinates, it was possible to track the geo-location of the Nordic walking session, as can be seen in Figure 9a,b. Free tools available on the web allowed creating a nice map with the followed path and returned the altimetry level encountered during the session.

The universal date and time available on the GPS signal played a key role in the post-processing synchronization algorithm since the two devices on the poles were not able to communicate wirelessly to each other during the tests.

### 3.4. Heart Rate Data

**Figure 10:**
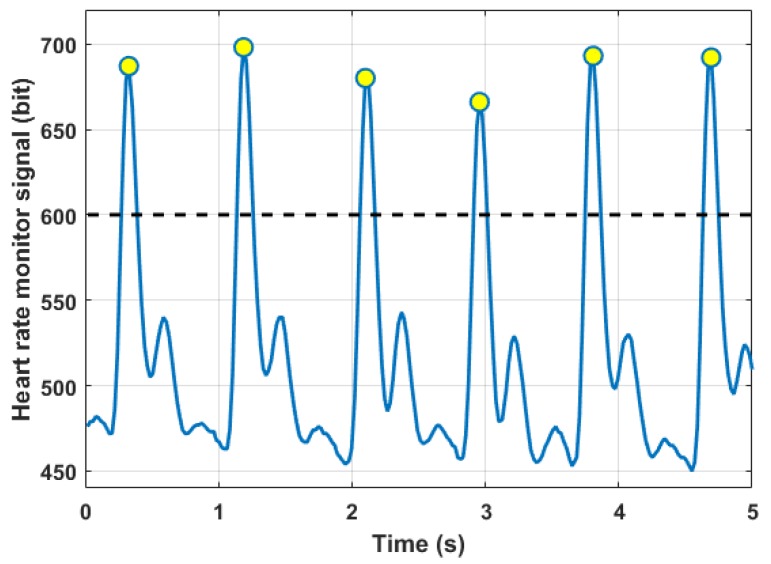
Heart rate sensor signal for BPM evaluation.

In Figure 10, it is possible to see a typical output signal of the heart rate sensor. Values on the y-axis are the digital representation of the sensor analogue output. A higher voltage is registered when the blood pressure wave passes below the light sensor. The number of Beats Per Minute (BPM) can be easily calculated by means of a peak counting above a certain signal threshold.

Due to the intrinsic behavior of the sensor, the signal had a very high sensitivity to noise components coming from the movement. Measurements were taken only when the user was still, for example in short pauses during the session. A future version of the data acquisition system should consider also different technologies to measure this parameter, leading to a better understanding during all of the workout session.

## 4. Acceleration and Force Measurements during the Walking Cycle

**Figure 11:**
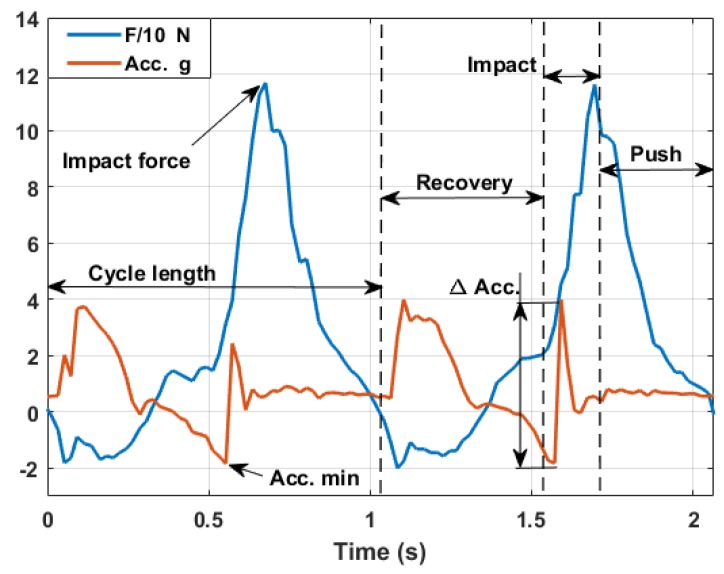
Acceleration-force comparison.

The adoption of an axial load cell would be the perfect solution for a complete view of each arm’s performance. Although several less expensive solutions to measure the pushing force may be exploited from a commercial perspective, here the authors want to highlight the relation between the peak force and the acceleration measured just before the impact as a possible solution. Comparing acceleration and force data (Figure 11), there is always a correspondence between the time when the force starts to rapidly increase and the acceleration measurement of the impact. According to Newton’s second law, there is a relation between acceleration and force. However, it is not so immediate to evaluate the inertial mass involved. In fact, the pole mass is just a fraction of the inertia contribution that will determine the total applied impact force. The equivalent contribution of the arm and hand should be also taken into account. Since it is not reasonable to measure these quantities for each user, the most suitable way is to establish a relation with the main human body parameters (height, weight and age) with the specific training level, body fat level and lean muscle mass. In Table 2, the results of such a kind of analysis are shown. Considering data coming from the same user, a detection algorithm identified 20 samples over a 2-min session as valid signals. This low number of samples is strongly related to the limited sampling frequency of the acquisition board. For future works focused on this type of analysis, it is advisable to consider higher sampling frequencies.

It must be noted that the measured acceleration is indeed the combination of two components:

(3) $$a_{x}=a_{s}+a_{d}$$

where:

- $a_{x}$ is the measured acceleration along the longitudinal pole axis;
- $a_{s}$ is the static component of the gravity acceleration;
- $a_{d}$ is the dynamic component related to the pole movement/impact.

To estimate the peak force, only the dynamic part of the acceleration should be taken into account. As a first approximation, considering that the pole angle does not have a large variation during the pushing phase, it is possible to evaluate $a_{d}$ in post-processing as:

(4) $$a_{d}=9.81\left(a_{x}-cos\left(\bar{\phi }\right)\right)$$

with $a_{x}$ the acceleration peak related to the impact ($a_{x}>0$) and $\bar{\phi }$ the mean angle of the following pushing phase. Keeping these considerations in mind, it is possible to have a good estimation of the peak force simply using accelerometer information if a proper statistical analysis is developed on several user typologies.

## 5. Conclusions

In this work, a specifically-designed monitoring system for Nordic walking applications is shown. The board had a set of sensors that allowed having a good picture of the user performance. It was capable of acquiring and saving data with an acquisition frequency of 50 Hz, fast enough to capture the relevant dynamics of the pole movement. The achieved performance was the best solution in terms of data acquisition frequency and energy consumption, allowing for long monitoring sessions. The use of two separate GPS sensors allowed both tracking the user during the session and synchronizing the two independent devices, allowing for data fusion and comparison. The left and right board were equipped with two different sensors: on the left side, a heart rate sensor monitored the heart rate of the user, giving a picture of the effort during the practice; on the right side, an axial load cell gave deeper insight into the pushing phase. Thanks to the fusion of the measurements coming from these sensors, it was possible to explore the performance from several points of view. The arm cycle frequency was monitored, understanding how long each single phase lasted compared to the whole cycle. Comparing left and right arm acceleration data, the adopted technique can be identified. The axial force represented important additional information giving deeper insight into the pushing phase. It was able to highlight the peak of the pushing force and gave an overview about how long and with what intensity the user relied on the pole during the activity. A comparison with studies available in the literature showed a good agreement between the measured quantities and data obtainable with indoor equipment. Thus, the system can be considered as an alternative measurement device for outdoor Nordic walking activities. The point of this work was to propose a device with the minimum impact possible on the user’s movements. Greater efforts can lead to improvements in the miniaturization of the prototype version in order to obtain the best performance-weight trade off. The gathered force and acceleration measurements will be used in future works as a validation for a multibody model of a Nordic walker. This mathematical model will be used to explore load distribution on joints during the practice. Finally, since the system can be implemented for commercial use, a simple procedure to estimate the peak force from accelerometer measurements was proposed. This method is pursuable only if a proper statistical data analysis is developed on several users with certain characteristics. Thus, the inertial constant can become the representation of a group of users.

## Figures and Tables

**Table 1:** Hardware components of the monitoring system.

Component	Device Name	Description
Accelerometer	ADXL345	Acceleration x, y, z
GPS	MTK3339	Position, data, time
Load cell	KM26z-500N	Force along *x* axis
Heart rate monitor	Pulse sensor Amped	Beats Per Minute (BPM)
Memory	μSD Card	Data storage
Microcontroller	ATMEGA 328P	Device management

**Table 2:** Inertia constant statistical evaluation.

Number of Samples	Mean Value (kg)	Standard Deviation (kg)
20	3.6	0.3
